# Iterated Clique Reductions in Vertex Weighted Coloring for Large Sparse Graphs

**DOI:** 10.3390/e25101376

**Published:** 2023-09-24

**Authors:** Yi Fan, Zaijun Zhang, Quan Yu, Yongxuan Lai, Kaile Su, Yiyuan Wang, Shiwei Pan, Longin Jan Latecki

**Affiliations:** 1 School of Mathematics and Statistic, Qiannan Normal University for Nationalities, Duyun 558000, China; yifan.sysu@gmail.com (Y.F.); quanyu@sgmtu.edu.cn (Q.Y.); 2 Key Laboratory of Complex Systems and Intelligent Optimization of Guizhou Province, Duyun 558000, China; 3 School of Mathematics and Information Engineering, Longyan University, Longyan 364000, China; laiyx@lyun.edu.cn; 4 Institute for Integrated and Intelligent Systems, Griffith University, Brisbane, QLD 4111, Australia; k.su@griffith.edu.au; 5 School of Computer Science and Information Technology, Northeast Normal University, Changchun 130024, China; wangyy912@nenu.edu.cn (Y.W.); pansw779@nenu.edu.cn (S.P.); 6 Department of Computer and Information Sciences, Temple University, Philadelphia, PA 19122, USA; latecki@temple.edu

**Keywords:** vertex weighted coloring, graph reduction, discrete optimization, clique sampling

## Abstract

The Minimum Vertex Weighted Coloring (MinVWC) problem is an important generalization of the classic Minimum Vertex Coloring (MinVC) problem which is NP-hard. Given a simple undirected graph G=(V,E), the MinVC problem is to find a coloring s.t. any pair of adjacent vertices are assigned different colors and the number of colors used is minimized. The MinVWC problem associates each vertex with a positive weight and defines the weight of a color to be the weight of its heaviest vertices, then the goal is the find a coloring that minimizes the sum of weights over all colors. Among various approaches, reduction is an effective one. It tries to obtain a subgraph whose optimal solutions can conveniently be extended into optimal ones for the whole graph, without costly branching. In this paper, we propose a reduction algorithm based on maximal clique enumeration. More specifically our algorithm utilizes a certain proportion of maximal cliques and obtains lower bounds in order to perform reductions. It alternates between clique sampling and graph reductions and consists of three successive procedures: promising clique reductions, better bound reductions and post reductions. Experimental results show that our algorithm returns considerably smaller subgraphs for numerous large benchmark graphs, compared to the most recent method named RedLS. Also, we evaluate individual impacts and some practical properties of our algorithm. Furthermore, we have a theorem which indicates that the reduction effects of our algorithm are equivalent to that of a counterpart which enumerates all maximal cliques in the whole graph if the run time is sufficiently long.

## 1. Introduction

Below we will introduce the MinVWC problem, current reduction approaches, and our proposed approach, together with some high-level motivation and comparisons.

### 1.1. The Problem

Given a simple undirected graph G=(V,E), a feasible coloring for *G* is an assignment of colors to *V* s.t. any pair of adjacent vertices are assigned different colors. Formally a *feasible coloring S* for G=(V,E) is defined as a partition $S=\{V_{1},\cdots ,V_{k}\}$ of *V* s.t. $V_{i}\neq \emptyset$ for any 1≤i≤k, $V_{i}\cap V_{j}=\emptyset$ for any 1≤i≠j≤k, ${⋃}_{i=1}^{k}V_{i}=V$, and for any edge {u,v}∈E, *u* and *v* are not in the same vertex subset $V_{i}$ where 1≤i≤k. Notice that *k* is unknown until we find a feasible coloring. In the Minimum Vertex Weighted Coloring (MinVWC) problem, each vertex is associated with a positive weight, i.e., there is an additional weighting function $w:V\mapsto Z^{+}$, and the goal is to find a feasible coloring that minimizes $\mathrm{cost}\left(S,G\right)=\sum_{i=1}^{k}\max_{v\in V_{i}}w\left(v\right)$. Obviously, an instance of the NP-hard MinVC problem can conveniently be reduced to an instance of the MinVWC problem by associating a weight of 1 with each vertex. As a result, the MinVWC problem is also NP-hard [1,2]. This problem arises in several applications like traffic assignment [3,4], manufacturing [5], scheduling [6] etc. Up to now, there are two types of algorithms for this problem: complete algorithms [3,7,8] and incomplete ones [4,9,10].

### 1.2. Current Reduction Approaches

In MinVC solving, a clique provides a lower bound for reductions because any two vertices in a clique cannot have the same color. In MinVWC solving, a clique is also able to do so, as can be found in the most recent reduction method RedLS published in [11]. Roughly it is desirable that we have cliques in hand that are of great sizes and each vertex in them has a big weight. So one may think that we can call an incomplete maximum vertex weight clique solver like [12,13] to obtain a list of optimal or near-optimal cliques. Such examples can be found in the state-of-the-art method RedLS. In detail, RedLS first performs reduction to obtain a reduced subgraph and then does a local search on that subgraph. In this paper, we will abuse the name RedLS to refer to its reduction component as well. As to its reduction component, RedLS first samples a proportion of vertices, and for each of them namely *v*, it tries to find one maximum or near-maximum vertex weight clique that contains *v*. Second it combines such cliques to obtain a ‘relaxed’ partition set and apply this set for reductions. In a nutshell, the reduction method of RedLS performs clique sampling and graph reduction successively without interleaving, which we believe is not so flexible and may miss a few promising cliques and bounds.

### 1.3. Our Approach

We do not believe that sampling maximum or near-maximum vertex weight cliques is a perfect approach for clique reductions. In fact, there are two types of cliques that may not have great total vertex weights but are still useful: those only with big size and those only with high-weight vertices, because they also contribute to a bound. Actually, solving MinVWC requires diversification, to be specific, a list of cliques that vary in both sizes and vertex weight distributions is preferred. If we call a maximum vertex weight clique solving procedure, we may finally obtain a list of cliques that lack such diversification, which results in relatively ineffective reductions. Therefore in this paper, we abandon such an approach and instead enumerate diverse cliques. In this sense enumerating all maximal cliques in the input graph seems to be a good choice, however, doing so may be costly and thus infeasible even in sparse graphs, so we develop an algorithm that only enumerates a certain proportion of them *but leads to equally effective reductions as the counterpart which enumerates all of them, if our algorithm completes*.

Recently complex networks have presented a number of applications like cloud computing [14,15], so research about vertex-weighted coloring in large complex networks is capturing great interest. In this paper, we will present a reduction algorithm that processes large sparse graphs in order to speed up current MinVWC solving. Roughly speaking, it alternates between clique sampling and graph reductions. In a graph reduction procedure, it obtains a subgraph whose optimal solutions can be extended into optimal ones for the whole graph, and we call this subgraph a VWC-reduced subgraph (Vertex Weighted Coloring-reduced subgraph). Since most large sparse graphs obey the power-distribution law [16,17], they can be reduced considerably by cliques of a certain quality. On the other hand, a smaller graph presents smaller search space and the algorithm may find better cliques more easily which can then be used for further reductions.

Our algorithm consists of three successive procedures. Firstly, we collect vertices that have maximum degrees or weights and enumerate all maximal cliques containing them. Each time we find a maximal clique we check whether it leads to further reductions and do so *immediately* if possible. Secondly, we systematically look for cliques that can trigger more effective reductions. As in the previous procedure, we will perform reductions immediately once we have found such a clique. Thirdly, we perform clique reductions which are ignored in the first two procedures. We evaluated our algorithm on a list of large sparse graphs that were accessed via http://networkrepository.com/ on 1 January 2018, and compared its performance with RedLS. Experimental results show that our reduction algorithm often returns subgraphs that are considerably smaller than those obtained by RedLS. Also, we evaluated the individual impacts of the three procedures above, and found that they all had significant contributions. Furthermore, our algorithm was able to confirm that it had found the best bound on a list of benchmark graphs. Last we have a theorem that indicates that although our algorithm only samples *a certain proportion of* maximal cliques in the whole graph, its reduction effects are equivalent to that of a counterpart that enumerates *all* of them in the whole graph, given sufficient run time.

## 2. Preliminaries

In what follows, we suppose a vertex weighted graph G=(V,E,w(·)) with $w:V\mapsto Z^{+}$ being a weighting function. If e={u,v} is an edge of *G*, we say that *u* and *v* are adjacent/connected and thus neighbors. Given a vertex *v*, we define the set of its neighbors, denoted by N(v), as {u∈V|{u,v}∈E} and we use N[v] to denote N(v)∪{v}. The degree of a vertex *v*, denoted by d(v), is defined as |N(v)|. A clique *C* is a subset of *V* s.t. any two vertices in *C* are mutually connected. A clique is said to be maximal if it is not a subset of any other clique. By convention we define size of a clique *C*, denoted by |C|, to be the number of vertices in it. Given a graph *G* and a vertex subset $V^{\prime }\subseteq V$, we use $G[V^{\prime }]$ to denote the subgraph of *G* which is induced by $V^{\prime }$, i.e., $G\left[V^{\prime }\right]=\left(V^{\prime },E^{\prime }\right)$ where $E^{\prime }=\left\{\left\{u,v\right\}\in E|u,v\in V^{\prime }\right\}$. Given a graph *G*, we use V(G) and E(G) to denote the set of vertices and edges of *G*, respectively.

In the following, for the ease of discussions, we generalize the notion of a coloring and allow it to color vertices not in *V*, so a coloring has now been redefined as $S=\{\langle 1,V_{1}\rangle ,\ldots ,\langle k,V_{k}\rangle \}$ with ${⋃}_{i=1}^{k}V_{i}⊇V$. Then we say that $V_{1},\ldots ,V_{k}$ are color classes and we redefine cost(S,G) as $\sum_{i=1}^{k}\max_{v\in V_{i}\cap V}w\left(v\right).$ Obviously according to new definitions, one coloring can have several representations, e.g., {〈1,U〉,〈2,V〉,〈3,W〉} and {〈1,W〉,〈2,V〉,〈3,U〉} represent the same coloring.

Given a graph *G*, we use ${S|}_{G}$ to denote a certain coloring for it. Then Proposition 1 below shows that given any feasible coloring, its cost on any induced subgraph does not exceed that on the whole graph.

**Proposition 1.**  *Suppose G=(V,E,w(·)) and U⊆V. If ${S|}_{G}$ is a feasible coloring for G, then*

1. *${S|}_{G}$ is also a feasible coloring for G[U];*
2. *$\mathrm{cost}\left(S{|}_{G},G\left[U\right]\right)\leq \mathrm{cost}\left(S{|}_{G},G\right)$.*

**Proof.**  See Appendix A.    □

Throughout this paper, when we say an optimal coloring/solution, we mean a feasible coloring/solution with the minimum cost. Given a vertex *u*, we use $c_{u}$ to denote *u*’s color. In addition, we use $c_{u}\leftarrow j$ to denote the operation which assigns *u* the color *j*, so $c_{u}\leftarrow c_{v}$ assigns *u* a color which is equal to that of *v*, i.e., which puts *u* in the same vertex subset with *v*.

Given a tuple $t=\langle x_{1},\cdots ,x_{l}\rangle$, we use |t| to denote the number of components in *t*, so |t|=l. For ease of expression, if *t* is an *empty* tuple, we define |t| to be 0. Given a map f:X↦Y and an element x∈X, if y=f(x), then we say that *y* is *x*’s image under *f* or simply say f(x) is *x*’s image under *f*. Such notions will be useful when we discuss the removal of vertices in clique reductions. Finally, when given vertices *u* and *v*, we say that *u* is *heavier* (resp. *lighter*) than *v* if w(u)>w(v) (resp. w(u)<w(v)).

### 2.1. A Reduction Framework

Below we will present notions that are related to graph reductions for the MinVWC problem. The first is an extension to a coloring which relates solutions for a subgraph to that for the whole graph.

**Definition 1.**  *Given a coloring $S=\{\langle 1,V_{1}\rangle ,\cdots ,\langle k,V_{k}\rangle \}$ and a vertex x s.t. $x\notin {⋃}_{i=1}^{k}V_{i}$, we define an extension to S with respect to $\left(c_{x}\leftarrow j\right)\left(1\leq j\leq k+1\right)$, denoted by $S⊎(c_{x}\leftarrow j)$, as*

$$S⊎\left(c_{x}\leftarrow j\right)=\left\{\begin{array}{cc} S\backslash \left\{\langle j,V_{j}\rangle \right\}\cup \left\{\langle j,V_{j}\cup \left\{x\right\}\rangle \right\} & \mathrm{if}1\leq j\leq k;\\ S\cup \{\langle j,\{x\}\rangle \} & \mathrm{if}j=k+1. \end{array}\right.$$

We also define *S* as an extension of itself. So an extension to *S* will not change the color of any vertices that have already been colored before. Instead, it will put a new vertex into one of the *k* existing vertex partitions if 1≤j≤k, or a new one if j=k+1. Obviously given two operations $a_{1}$ and $a_{2}$ for extensions, we have $\left(S⊎a_{1}\right)⊎a_{2}=\left(S⊎a_{2}\right)⊎a_{1}$, so the order of the operations does not matter.

Given a set $A=\{a_{1},\ldots ,a_{n}\}$, we use S⊎A to denote $S⊎a_{1}⊎\ldots ⊎a_{n}$, and we also say S⊎A is an extension to *S*. Last if S⊎A or $S⊎(c_{x}\leftarrow j)$ is a feasible color for *G*, then we say that S⊎A or $S⊎(c_{x}\leftarrow j)$ is a feasible extension to *S* for *G*. Below we have a proposition that will be useful in proving other later propositions.

The proposition below illustrates that extending a coloring will not decrease its cost.

**Proposition 2.**  *Given a vertex x∈V and a coloring $S=\{\langle 1,V_{1}\rangle ,\cdots ,\langle k,V_{k}\rangle \}$ for G[V∖{x}], then*

$$\mathrm{cost}\left(S,G\left[V\setminus \left\{x\right\}\right]\right)\leq \mathrm{cost}\left(S⊎\left(c_{x}\leftarrow j\right),G\right)$$

*for any 1≤j≤k+1.*

**Proof.**  See Appendix B.    □

Next, we define a type of subgraphs whose feasible solutions can be extended into feasible ones for the whole graph with the same cost.

**Definition 2.**  *Suppose G=(V,E,w(·)) and U⊆V. If given any feasible coloring ${S|}_{G[U]}$ for G[U], there exists an extension to ${S|}_{G[U]}$, denoted by ${S|}_{G}$, such that ${S|}_{G}$ is feasible for G and $\mathrm{cost}\left(S{|}_{G[U]},G\left[U\right]\right)=\mathrm{cost}\left(S{|}_{G},G\right)$, then we say that G[U] is a VWC-reduced subgraph for G.*

This notion of VWC-reduced subgraph has two nice properties which are shown in Propositions 3 and 4 below. In detail, Proposition 3 shows that the relation of the VWC-reduced subgraph is transitive and we can compute a VWC-reduced subgraph in an iterative way.

**Proposition 3.**  *Suppose G=(V,E,w(·)), W⊆U⊆V, G[W] is a VWC-reduced subgraph for G[U] and G[U] is a VWC-reduced subgraph for G, then G[W] is a VWC-reduced subgraph for G.*

**Proof.**  See Appendix C.    □

Proposition 4 shows that in order to find an optimal solution for *G*, we can first find an optimal solution for its VWC-reduced subgraphs.

**Proposition 4.**  *Suppose G=(V,E,w(·)), U⊂V and G[U] is a VWC-reduced subgraph of G, then*

*1.*  given any optimal feasible solution $S^{*}{|}_{G[U]}$ for G[U], there exists an extension to $S^{*}{|}_{G[U]}$ which is an optimal solution for G;
*2.*  *given any non-optimal feasible solution $S^{↓}{|}_{G[U]}$ for G[U], there exist no extension to $S^{↓}{|}_{G[U]}$ that is an optimal solution for G.*

**Proof.**  See Appendix D.    □

These propositions allow our algorithms to interleave between clique sampling and graph reduction, which is different from the approach in RedLS [11] yet similar to that in FastWClq [12]. This is why we titled this paper ‘iterative clique reductions’.

In what follows we will introduce a general principle for computing VWC-reduced subgraphs.

### 2.2. Clique Reductions

Below we will utilize the notion of VWC-reduced subgraph to introduce clique reductions which was initially proposed in [11]. First, we introduce the notion of *absorb* which illustrates that a vertex’s close neighborhood is a weak sub-structure of a clique.

**Definition 3.**  *Given a vertex u and a clique $C=\{v_{1},\cdots ,v_{\left|C\right|}\}$ in G s.t. u∉C, |C|>d(u), $w\left(v_{1}\right)\geq \cdots \geq w\left(v_{\left|C\right|}\right)$ and $w\left(v_{d(u)+1}\right)\geq w\left(u\right)$, then we say that u is absorbed by C.*

Note that the condition |C|>d(u) guarantees that $w(v_{d(u)+1})$ always exists. Also notice that [11] did not allow the equation in $w\left(v_{d(u)+1}\right)\geq w\left(u\right)$ to hold, but we extend their statements slightly.

**Example 1.**  *Consider $G_{1}$ in which $z_{i}^{\omega }$ denotes Vertex $z_{i}$ with a weight ω. Let $u=z_{4}^{2}$ and $C=\{z_{5}^{6},z_{2}^{5},z_{6}^{4},z_{1}^{3}\}$, then u∉C, |C|=4 and d(u)=3, thus |C|>d(u) and $w\left(v_{d(u)+1}\right)=w\left(z_{1}^{3}\right)=3\geq w\left(u\right)=2$. So we say that $z_{4}^{2}$ is absorbed by C.*

*To make our descriptions more intuitive, we show C and N[u] separately below and moreover, in C a heavier vertex is shown in a darker color. If we left-shift N[u], then we will find that there is a one-to-one map ξ:N[u]↦C namely $\left\{\langle z_{4}^{2},z_{1}^{3}\rangle ,\langle z_{3}^{5},z_{2}^{5}\rangle ,\langle z_{7}^{1},z_{5}^{6}\rangle ,\langle z_{8}^{5},z_{6}^{4}\rangle \right\}$, s.t.*

*1.*  *w(u)≤w(ξ(u)), that is, u is no heavier than its image under ξ;*
*2.*  *and for any x∈N(u), w(ξ(x))≥w(ξ(u)), that is, images of u’s neighbors are no lighter than that of u, or we may roughly say that u’s image is the lightest compared to those of its neighbors.*


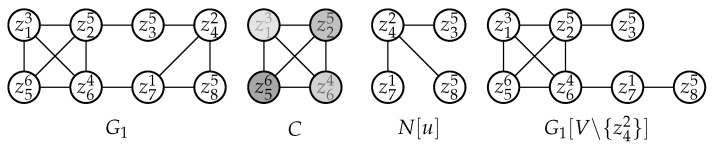

*Since |C|=4, there exist at least 4 colors in any feasible solution for $G_{1}\left[V\setminus \left\{u\right\}\right]$. For coloring vertices in N(u), we only need d(u)=3 colors, so there exists at least one color among that of $z_{5}^{6},z_{2}^{5},z_{6}^{4},z_{1}^{3}$ which is not in use for N(u), and we can use it to color u namely $z_{4}^{2}$ without causing any conflicts. Because $w\left(z_{1}^{3}\right)\geq w\left(z_{4}^{2}\right)$, even though we assign $z_{4}^{2}$ the same color as that of $z_{1}^{3}$, the lightest vertex in C, the cost of that coloring will not increase. So we can now simply ignore $z_{4}^{2}$ and later assign it an existing color after all its neighbors have been colored, depending on its weight as well as its neighbors’ colors. Obviously, this is a feasible extension that does not increase the cost of a coloring. Therefore $G_{1}\left[V\setminus \left\{z_{4}^{2}\right\}\right]$ is a VWC-reduced subgraph of $G_{1}$.*

In general, we have a proposition below [11].

**Proposition 5.**  *Given a graph G and a vertex u, if there exists a clique C s.t. u is absorbed by C, then G[V∖{u}] is a VWC-reduced subgraph of G.*

**Proof.**  See Appendix E.    □

So if a vertex is absorbed by a clique, it can be removed in order to obtain a VWC-reduced subgraph.

**Example 2.**  *Now we continue with Example 1.*

*1.*  *In $G_{1}\left[V\setminus \left\{z_{4}^{2}\right\}\right]$, we find that $z_{8}^{5}$ is absorbed by C, so we have $G_{1}\left[V\setminus \left\{z_{4}^{2},z_{8}^{5}\right\}\right]$ is a VWC-reduced subgraph of $G_{1}\left[V\setminus \left\{z_{4}^{2}\right\}\right]$. Similarly we have $G_{1}\left[V\setminus \left\{z_{4}^{2},z_{8}^{5},z_{3}^{5}\right\}\right]$ is that of $G_{1}\left[V\setminus \left\{z_{4}^{2},z_{8}^{5}\right\}\right]$ and $G_{1}\left[V\setminus \left\{z_{4}^{2},z_{8}^{5},z_{3}^{5},z_{7}^{1}\right\}\right]$ is that of $G_{1}\left[V\setminus \left\{z_{4}^{2},z_{8}^{5},z_{3}^{5}\right\}\right]$.*
*2.*  *By Proposition 3, we have $G_{1}\left[V\setminus \left\{z_{4}^{2},z_{8}^{5},z_{3}^{5},z_{7}^{1}\right\}\right]$ is that of $G_{1}$. Also we have an optimal coloring for $G_{1}\left[V\setminus \left\{z_{4}^{2},z_{8}^{5},z_{3}^{5},z_{7}^{1}\right\}\right]$ is*
$$S^{*}{|}_{G_{1}\left[V\setminus \left\{z_{1}^{3},z_{2}^{5},z_{5}^{6},z_{6}^{4}\right\}\right]}=\left\{\langle 1,\left\{z_{1}^{3}\right\}\rangle ,\langle 2,\left\{z_{2}^{5}\right\}\rangle ,\langle 3,\left\{z_{5}^{6}\right\}\rangle ,\langle 4,\left\{z_{6}^{4}\right\}\rangle \right\}$$
and $\mathrm{cost}(S^{*}{|}_{G_{1}\left[V\setminus \left\{z_{4}^{2},z_{8}^{5},z_{3}^{5},z_{7}^{1}\right\}\right]},G_{1}\left[V\setminus \left\{z_{4}^{2},z_{8}^{5},z_{3}^{5},z_{7}^{1}\right\}\right])=3+5+6+4=18$.
*3.*  Considering Proposition 4, there exists a feasible extension to $S^{*}{|}_{G_{1}\left[V\setminus \left\{z_{4}^{2},z_{8}^{5},z_{3}^{5},z_{7}^{1}\right\}\right]}$, denoted by $S^{*}{|}_{G_{1}}$, s.t. $S^{*}{|}_{G_{1}}$ is an optimal solution for $G_{1}$. In detail, for coloring the removed vertices in $\left\{z_{4}^{2},z_{8}^{5},z_{3}^{5},z_{7}^{1}\right\}$, we can follow the reversed order of the reductions before.
*4.*  *So an optimal coloring for $G_{1}$ is*
$$S^{*}{|}_{G_{1}}=\left\{\langle 1,\left\{z_{1}^{3}\right\}\rangle ,\langle 2,\left\{z_{2}^{5},z_{8}^{5}\right\}\rangle ,\langle 3,\left\{z_{5}^{6},z_{3}^{5},z_{7}^{1}\right\}\rangle ,\langle 4,\left\{z_{6}^{4},z_{4}^{2}\right\}\rangle \right\}$$
and $\mathrm{cost}(S^{*}{|}_{G_{1}},G_{1})=18$.

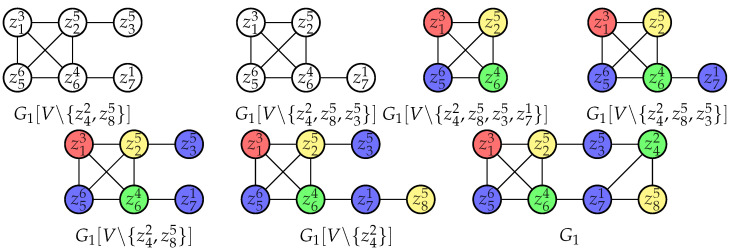

Furthermore we only have to focus on maximal cliques as is shown by the proposition below.

**Proposition 6.**  *If u is absorbed by a clique in G, then it must be absorbed by a maximal clique in G.*

From the propositions above, we can see that whether a vertex can be removed to obtain a VWC-reduced subgraph or not depends on the quality of the cliques in hand. Below we define a partial order ⊑ between cliques which indicates whether vertices absorbed by one clique are a subset of those absorbed by the other.

**Definition 4.**  *Given a graph G=(V,E,w(·)) and its two cliques $C_{x}=\{x_{1},\cdots ,\;$$x_{|C_{x}|}\}$ and $C_{y}=\left\{y_{1},\cdots ,\;y_{|C_{y}|}\right\}$ where $w\left(x_{1}\right)\geq \cdots \geq w\left(x_{|C_{x}|}\right)$ and $w\left(y_{1}\right)\geq \cdots \geq w\left(y_{|C_{y}|}\right)$, we define a partial order ⊑ s.t. $C_{x}⊑C_{y}$ iff*

*1.*  *$|C_{x}\left|\leq \right|C_{y}|$;*
*2.*  *$w\left(x_{t}\right)\leq w\left(y_{t}\right)$ for $1\leq t\leq |C_{x}|$.*

So if $C_{x}⊑C_{y}$, then $C_{y}$ leads to reductions that are at least as effective as that result from $C_{x}$. In what follows, if $C_{x}⊑C_{y}$, we say that *$C_{x}$ is subsumed by $C_{y}$*. Obviously we have a proposition below which shows that the ⊑ relation is transitive.

**Proposition 7.**  *Given a graph G=(V,E,w(·)) and its three cliques $C_{x},C_{y},C_{z}$, if $C_{x}⊑C_{y}$ and $C_{y}⊑C_{z}$, then $C_{x}⊑C_{z}$.*

Then we have two propositions which show that if $C_{x}⊑C_{y}$, then we can keep $C_{y}$ and ignore $C_{x}$.

**Proposition 8.**  *Suppose u is a vertex and $C_{x},C_{y}$ are cliques s.t. $u\notin C_{x}\cup C_{y}$ and $C_{x}⊑C_{y}$, then if u is absorbed by $C_{x}$, then it is also absorbed by $C_{y}$.*

The proposition below states that if there occur reductions among $C_{x}$, $C_{y}$ and their vertices where $C_{x}⊑C_{y}$, then keeping $C_{y}$ is at least as good as keeping $C_{x}$.

**Proposition 9.**  *Suppose $C_{x},C_{y}$ are cliques s.t. $C_{x}=\{x_{1},\cdots ,\;$$x_{|C_{x}|}\}$ and $C_{y}=\left\{y_{1},\cdots ,\;y_{|C_{y}|}\right\}$ where $w\left(x_{1}\right)\geq \cdots \geq w\left(x_{|C_{x}|}\right)$ and $w\left(y_{1}\right)\geq \cdots \geq w\left(y_{|C_{y}|}\right)$, if $C_{x}\cap C_{y}=\emptyset$ and $C_{x}⊑C_{y}$, then we have for any $1\leq t\leq |C_{x}|$, if $y_{t}$ is absorbed by $C_{x}$, then $x_{t}$ is absorbed by $C_{y}$.*

So if we utilize $C_{x}$ and $C_{y}$ to perform clique reductions where $C_{x}⊑C_{y}$, we can simply ignore $C_{x}$ and keep $C_{y}$.

### 2.3. A State-of-the-Art Reduction Method

To date, as we know, the only work on reductions for vertex weighted coloring is RedLS [11], which constructs promising cliques like FastWClq [12] and combines these cliques in an appropriate way to obtain a ‘relaxed’ partition set. Then it utilizes this set to perform reductions and compute lower bounds. So RedLS consists of clique sampling and graph reductions as successive procedures without interleaving.

Notice that FastWClq alternates between clique sampling and graph reduction and it benefits much from this approach. Hence it will be interesting to try whether such an alternating approach would lead to better reductions in vertex weighted coloring. Fortunately, the reduction framework introduced above allows us to do so.

For simplicity, we will put the details of RedLS in Section 4, where we will be able to reuse our notations and algorithms for succinct presentation.

## 3. Our Algorithm

Our reduction algorithm consists of three successive procedures: Algorithms 1 and 2 and post reductions in Section 3.3. As to Algorithm 1, we will first run it with maximum-weight vertices assigned to startVertexSet in Line 1 and then run it again with maximum-degree vertices in the same way.

### 3.1. Sampling Promising Cliques

Algorithm 1 samples promising cliques that may lead to considerable reductions with three components as below.

1. startVertexSet contains maximum degree/weight vertices and helps find promising cliques.
2. criticalCliqSet contains cliques that may probably lead to effective reductions and will be utilized in post reductions in Section 3.3.
3. topLevelWeights is a list of weights in non-increasing order and will be used for reductions.

In Line 7, we adopt depth-first search to enumerate all maximal cliques which contain vertices only in candSet. This operation can be costly, so in Section 3.4, we will set a cutoff for it. To be specific, before each enumeration, we will first put all related vertices into a list and shuffle this list randomly, then we will pick decision vertices one after another in this list to construct maximal cliques. By decision vertices, we mean those vertices that can both be included and excluded to form different maximal cliques.

Furthermore, Lines 8, 9, 10, and 16 will be introduced in Definition 8. Lines 21 and 22 are based on Proposition 16 and will be introduced in detail there.
**Algorithm 1:** PromisingCliqueReductions
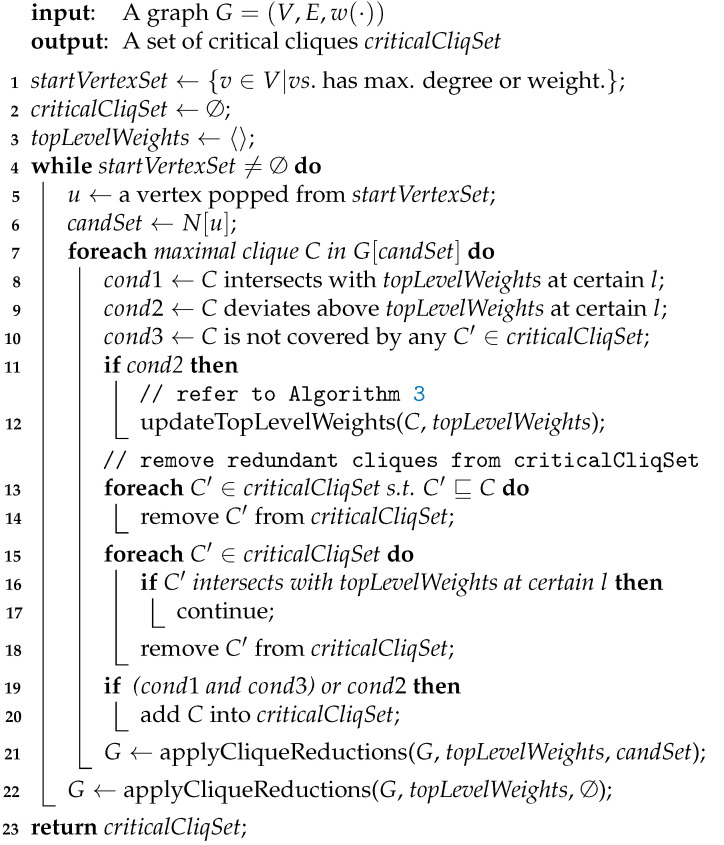


#### 3.1.1. Geometric Representations

First, we introduce a notation for representing weight distributions within given cliques.

**Definition 5.**  *Given a clique $C=\{v_{1},\cdots ,v_{\left|C\right|}\}$ s.t. C≠∅ where $w\left(v_{1}\right)\geq \cdots \geq w\left(v_{\left|C\right|}\right)$, we define its weight list, denoted by δ(C), to be $\langle w\left(v_{1}\right),\cdots ,w\left(v_{\left|C\right|}\right)\rangle$.*

Second, we introduce an operator for appending items to the end of a weight list, and it is somewhat like counterparts for vector in C++, ArrayList in Java, or list in Python.

**Definition 6.**  *Given a list of weights L and a weight ω, we define L⊕ω as 〈ω〉 if L=〈〉 and as $\langle \omega_{1},\cdots ,\omega_{t},\omega \rangle$ if $L=\langle \omega_{1},\cdots ,\omega_{t}\rangle$.*

In order to describe properties of our algorithms intuitively, we introduce Euclidean geometric representations of a list of weights in a rectangular coordinate system as below.
**Algorithm 2:** BetterBoundReductions
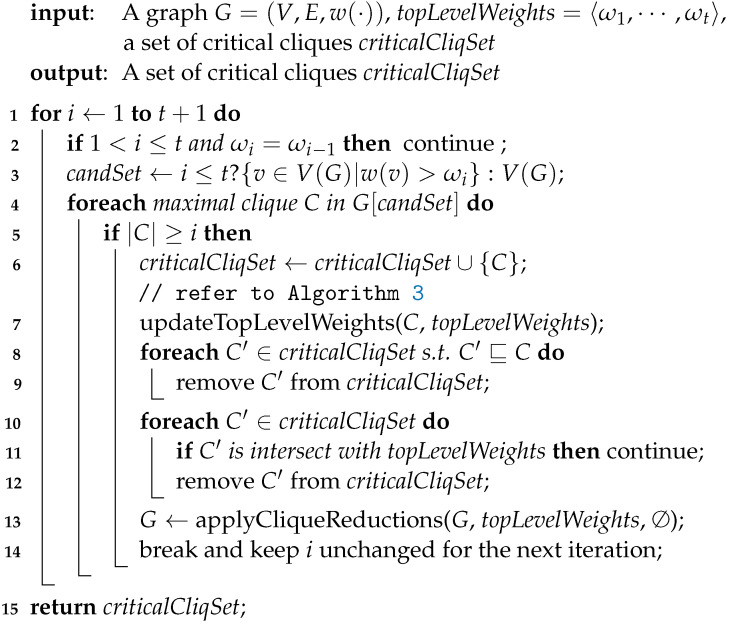


**Algorithm 3:** updateTopLevelWeights

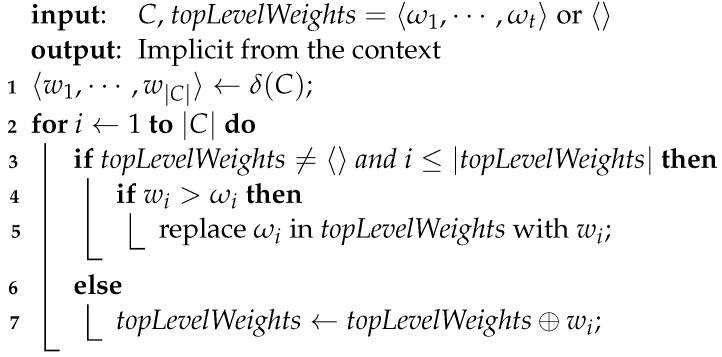

**Definition 7.**  *Given a list of positive numbers $L=\langle d_{1},\cdots ,d_{t}\rangle$, we draw a curve on the Rectangular Coordinate Plane xOy with the list of coordinates $\langle \left(1,d_{1}\right),\cdots ,\left(t,d_{t}\right)\rangle$ by connecting adjacent points, and we call this curve the derived curve of L.*

**Example 3.**  *Notice $G_{2}$. There are three maximal cliques, $C_{1}=\left\{z_{1}^{3},z_{2}^{4},z_{3}^{5},z_{4}^{1},z_{5}^{2}\right\}$, $C_{2}=\left\{z_{1}^{3},z_{4}^{1},z_{6}^{7},z_{5}^{2}\right\}$ and $C_{3}=\left\{z_{5}^{2},z_{4}^{1},z_{7}^{6},z_{6}^{7}\right\}$ with $\delta \left(C_{1}\right)=\langle 5,4,3,2,1\rangle$, $\delta \left(C_{2}\right)=\langle 7,3,2,1\rangle$ and $\delta \left(C_{3}\right)=\langle 7,6,2,1\rangle$.*


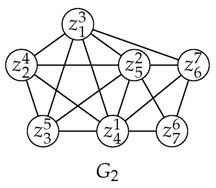

**Figure 1:**
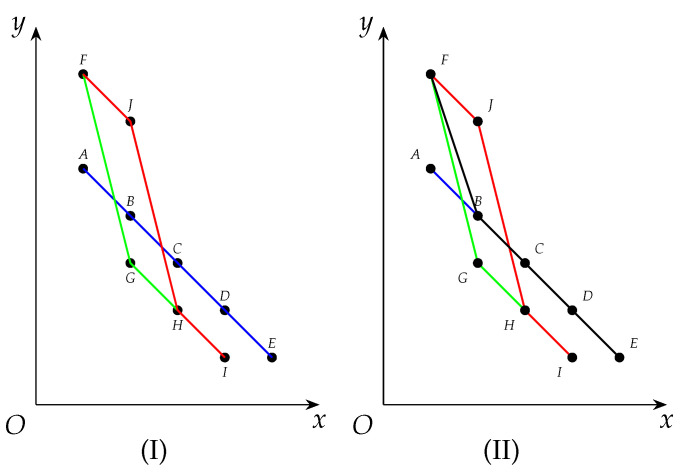
(**I**) Derived curves of $\delta (C_{1})$, $\delta (C_{2})$ and $\delta (C_{3})$ represented by blue, green and red curves, respectively; (**II**) topLevelWeights after being updated by $C_{1}$ and $C_{2}$ successively, which is the tightest envelope of them.

*We draw the derived curves of $\delta (C_{1})$, $\delta (C_{2})$ and $\delta (C_{3})$ as ABCDE (blue), FGHI (green) and FJHI (red) in (I) in Figure 1.*

On the other hand, we draw the derived curve of topLevelWeights which has just been updated with respect to $C_{1}$ and $C_{2}$ successively in Algorithm 3 as FBCDE (black) in (II) in Figure 1.

1. In detail, when topLevelWeights has just been updated with respect to $C_{1}$, its derived curve exactly overlaps that of $\delta (C_{1})$.
2. Next when topLevelWeights has just been updated with respect to $C_{2}$, a part of its derived curve, namely AB, has moved to its top-right, namely FB, so the derived curve of topLevelWeights has turned into FBCDE. Notice that having been updated with respect to $C_{1}$ and $C_{2}$, the derived curve of topLevelWeights is the bottom-left most curve that is not exceeded by that of $C_{1}$ and $C_{2}$. In other words, topLevelWeights has become the tightest envelope of that of $C_{1}$ and $C_{2}$.

Actually, if we switch the order of $C_{1}$ and $C_{2}$ in the procedure above, we will obtain the same sequence in topLevelWeights. In general, from the second time on, each time Algorithm 3 ends with topLevelWeights being updated, parts of the derived curve of topLevelWeights move to their top-right.

Now we consider the derived curves of topLevelWeights and δ(C) and define several notions below which describe the relationship between a vertex weighted clique and a list of non-increasing weights.

**Definition 8.**  *Given a list of weights $L=\langle \omega_{1},\cdots ,\omega_{t}\rangle$ s.t. $\omega_{1}\geq \cdots \geq \omega_{t}$ and a clique C with C≠∅ and $\delta \left(C\right)=\langle w_{1},\cdots ,w_{\left|C\right|}\rangle$,*

*1.*  *we say that C is covered by L iff t≥|C| and $\omega_{i}\geq w_{i}$ for any 1≤i≤|C|;*
*2.*  *we say that C intersects with L at l iff 1≤l≤min{|C|,t} and $w_{l}=\omega_{l}$;*
*3.*  *we say that C deviates above L at l iff $1\leq l\leq \min\left\{|C|,t\right\}\wedge w_{l}>\omega_{l}$ or |C|≥l>t.*

**Example 4.**  *Consider Example 3 with topLevelWeights having been updated with respect to $C_{1}$ and $C_{2}$. By referring to (II) in Figure 1, we can find the following.*

*1.*  *$C_{1}$ and $C_{2}$ are covered by topLevelWeights.*
*2.*  *$C_{1}$ intersects with topLevelWeights at 2, 3, 4 and 5 (see B, C, D and E). $C_{2}$ intersects with topLevelWeights at 1 (see F).*
*3.*  *$C_{3}$ deviates above topLevelWeights at 2 (see J).*

Obviously, we have a proposition below which helps determine whether a clique is effective in reductions.

**Proposition 10.** 

*1.*  *$C_{1}⊑C_{2}$ iff $\delta (C_{1})$ is covered by $\delta (C_{2})$.*
*2.*  *If $L_{1}$ is covered by $L_{2}$, $L_{2}$ is covered by $L_{3}$, then $L_{1}$ is covered by $L_{3}$.*
*3.*  *If $C_{1}$ deviates above L at certain l and $C_{2}$ is covered by L, then $C_{1}⋢C_{2}$.*

#### 3.1.2. Algorithm Execution

As to the execution of Algorithm 3, the next proposition presents a sufficient and necessary condition in which topLevelWeights will be updated.

**Proposition 11.**  *The topLevelWeights in Algorithm 3 will be updated if and only if topLevelWeights=〈〉 or the input clique C deviates above topLevelWeights at certain l.*

Also, we have propositions below which illustrate how topLevelWeights will be updated.

**Proposition 12** (First Top-level Insertion)**.** *Suppose that topLevelWeights=〈〉 and $\delta \left(C\right)=\langle w_{1},\cdots ,w_{\left|C\right|}\rangle$, then $w_{1},\cdots ,w_{\left|C\right|}$ will successively be appended to the end of topLevelWeights in Line 7 in Algorithm 3.*

**Proposition 13** (Successor Top-level Updates)**.** *Suppose $\mathrm{topLevelWeights}=\langle \omega_{1},\cdots ,\omega_{t}\rangle$ where t≥1,*

*1.*  *for any 1≤l≤t, $\omega_{l}$ will be replaced with $w_{l}$ in Line 5 in Algorithm 3 iff C deviates above topLevelWeights at l;*
*2.*  *for any l>t, a weight $w_{l}$ will be inserted in Line 7 in Algorithm 3 iff C deviates above topLevelWeights at l.*

The following proposition shows the relation between topLevelWeights and *C* if it has been updated in Algorithm 3.

**Proposition 14.**  *If topLevelWeights has been updated in Algorithm 3, then topLevelWeights covers the clique C at the end of this algorithm.*

Such a covering relation will still hold after Algorithm 3 returns program control back to Algorithm 1. Then we have a proposition about criticalCliqSet in Algorithm 1.

**Proposition 15.** 

*1.*  *Right before the execution of Line 22, for any $C^{\prime }\in \mathrm{criticalCliqSet}$, $C^{\prime }$ is covered by topLevelWeights.*
*2.*  *In Line 19, if C deviates above topLevelWeights, then for any $C^{\prime }\in \mathrm{criticalCliqSet}$, we have $C⋢C^{\prime }$.*

Intuitively right before the execution of Line 21, topLevelWeights can do whatever any clique in criticalCliqSet can, with exceptions being dealt with in Section 3.3. In Line 19, if *C* updates topLevelWeights, then it will be allowed an entry into criticalCliqSet.

**Figure 2:**
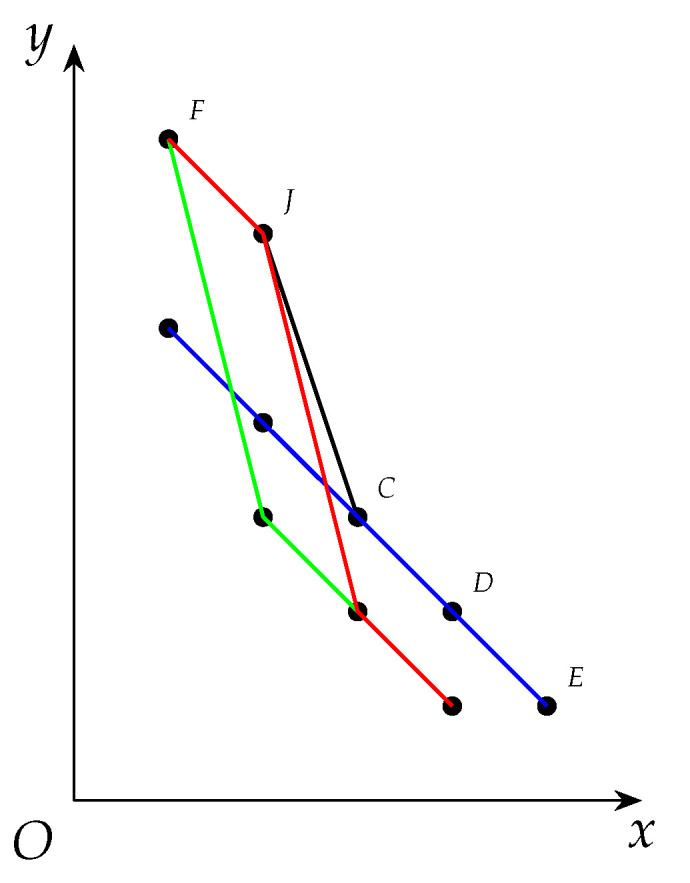
topLevelWeights after being updated by $C_{1}$, $C_{2}$ and $C_{3}$. Derived curves of $\delta (C_{1})$, $\delta (C_{2})$, $\delta (C_{3})$ and topLevelWeights represented by blue, green, red and black curves respectively.

**Example 5.**  *After Algorithm 1 is run on $G_{2}$ in Example 3, criticalCliqSet has become $\left\{C_{1},C_{3}\right\}$ and topLevelWeights has been updated to be 〈7,6,3,2,1〉, as is shown as FJCDE in Figure 2. The details are as follows.*

*1.*  *$C_{2}\notin \mathrm{criticalCliqSet}$ because $C_{2}⊑C_{3}$ but $C_{3}⋢C_{2}$. So either $C_{2}$ was refused to enter criticalCliqSet or it was removed from criticalCliqSet, depending on whether the algorithm found $C_{2}$ earlier than it found $C_{3}$.*
*2.*  *$C_{1}$ (blue) and $C_{3}$ (red) are both covered by topLevelWeights.*
*3.*  *As to the two cliques above, neither subsumes the other.*

So in Line 19, cond2 implies cond3. In other words, if cond2 holds, then *C* is not covered by any clique in criticalCliqSet, i.e., *C* is not *subsumed* by any clique in criticalCliqSet. In this sense, we add it to criticalCliqSet and this will not cause obvious redundancy.

Based on the discussion above, we have

1. right before the execution of Line 21 in Algorithm 1, topLevelWeights contains best-found bounds formed by all previous enumerated cliques;
2. and if any clique improves this bound, then no previously enumerated clique subsumes it. Unlike [11], we will apply topLevelWeights instead of ‘relaxed’ partition set to perform reductions in Algorithms 1 and 2.

Furthermore, for the sake of efficiency, we should keep criticalCliqSet as small as possible and as powerful as possible. So in Algorithm 1, if $C^{\prime }⊑C$, i.e., $C^{\prime }$ is subsumed by *C*, then we will simply remove $C^{\prime }$ in Line 14 and this will do no harm to the power of criticalCliqSet. In addition, if $C^{\prime }$ does not intersects with the derived curve of topLevelWeights, its reduction power is overwhelmed by topLevelWeights, so we remove it in Line 18 as well.

#### 3.1.3. Reductions Based on Top Level Weights

Next we have a proposition below which states that topLevelWeights can be utilized for clique reductions.

**Proposition 16.**  *Given $\mathrm{topLevelWeights}=\langle \omega_{1},\cdots ,$$\omega_{t}\rangle$, then*

*1.*  *for any 1≤l≤t, there exists a clique $Q=\{v_{1},\cdots ,v_{l}\}$ and $w\left(v_{1}\right)\geq \cdots \geq w\left(v_{l}\right)=\omega_{l}$;*
*2.*  *given any feasible coloring $S=\{\langle 1,V_{1}\rangle ,\cdots ,\langle k,V_{k}\rangle \}$ for G, k≥t;*
*3.*  *given any vertex u s.t. d(u)<t and $\omega_{d(u)+1}>w\left(u\right)$,*
  *(a)*  *u is absorbed by some certain clique in G;*
  *(b)*  *and G[V∖{u}] is a VWC-reduced subgraph of G.*

Notice that Item 1 states that topLevelWeights is the *tightest* envelope of all cliques that have been enumerated (See Figure 2 above for details and intuition).

1. In this sense, if *t* was decreased or any of $\omega_{1},\cdots ,\omega_{t}$ was decreased, the derived curve of topLevelWeights would be left or down shift, which in turn, made at least one clique deviate above topLevelWeights at some certain *l*. Therefore there must exist a color whose weight was smaller than its lower-bound.
2. Considering that weights of other colors are all underestimated, we have the sum of all components in the new variant of topLevelWeights could never be achieved by any feasible coloring.

So in order to obtain a feasible coloring that avoids lower-bound conflicts in any enumerated cliques, we have to accept the cost revealed by topLevelWeights or even more. In a word, any feasible coloring for *G* costs at least $\sum_{i=1}^{t}\omega_{t}$, which will be shown and proved formally in Proposition 17 and has also been proved by [11] in another approach.

**Figure 3:**
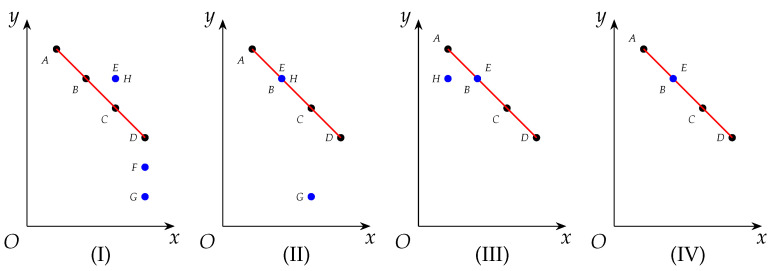
Iterated Removals. ABCD represents topLevelWeights while E,F,G,H represents $z_{3}^{5},z_{4}^{2},z_{7}^{1},z_{8}^{5}$ respectively. (**I**) Right before reductions; (**II**) $z_{4}^{2}$ been removed; (**III**) $z_{7}^{1}$ been removed; (**IV**) $z_{8}^{5}$ been removed.

Given a vertex *u*, we represent it as a point $P_{u}=\left(d\left(u\right)+1,w\left(u\right)\right)$ on the Rectangular Coordinate Plane xOy in order for intuition (See Figure 3). Then we have $P_{u}$*is strictly below the derived curve of*topLevelWeights*iff*d(u)<t*and*$\omega_{d(u)+1}>w\left(u\right)$, and such a location relation implies Items 3a and 3b above. Moreover each time one neighbor of *u* is removed, d(u) will be decreased by 1 and the point $P_{u}$ will be left shift by 1. Meanwhile, when we enumerate cliques, topLevelWeights tend to move to its top-right. These opposite trends will gradually help reduce the input graph.

**Example 6.**  *Consider $G_{1}$ in Example 1 in which there exists a maximal clique $C=\{z_{1}^{3},z_{2}^{5},z_{5}^{6},z_{6}^{4}\}$ with δ(C)=〈6,5,4,3〉. As to the four other vertices $z_{3}^{5},z_{4}^{2},z_{7}^{1},z_{8}^{5}$ with degrees 2,3,3,2, we represent them by E,F,G,H, respectively, on a rectangular coordinate plane in (I) in Figure 3 below. For instance, the coordinate of F is $\left(d\left(z_{4}^{2}\right)+1,w\left(z_{4}^{2}\right)\right)$ namely (4,2). Notice that $z_{3}^{5}$ and $z_{8}^{5}$ have the same degree and weight, so their corresponding points overlap on the coordinate plane, to be specific, $z_{3}^{5}$ and $z_{8}^{5}$ are represented by E and H, respectively, which overlap.*


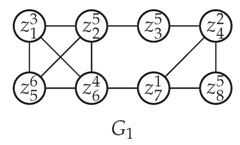

On the other hand, we can utilize topLevelWeights instead of specific cliques to perform clique reductions. For example, Line 21 in Algorithm 1 exploits topLevelWeights to perform reductions based on Proposition 16 above. In detail, G←applyCliqueReductions(*G*, topLevelWeights, *S*) performs clique reductions and obtain a VWC-reduced subgraph of *G*, but keeps all vertices in *S* in the returned subgraph. We do this for the following reason: In Line 21, since we are enumerating cliques in candSet, we should keep all vertices in it. Otherwise, the procedure may crash. However, in Line 22, since we have completed the enumeration procedures, we do not have to keep any vertices in the VWC-reduced subgraph. We also remind readers that in the applyCliqueReductions procedure, each time one vertex is removed, all its neighbors will be taken into account for further reductions because their degrees have all been decreased by 1.

Notice that in Proposition 16 we require $\omega_{d(u)+1}>w\left(u\right)$ rather than $\omega_{d(u)+1}\geq w\left(u\right)$, because we have to ensure that *u* is absorbed by a clique that does not contain *u*, which is coincident with the approach in [11]. However, this method may fail to perform some reductions which can be performed by Proposition 5. Yet this is not a problem, because, at the end of our reductions, we will deal with that case. See Section 3.3 for more details.

**Example 7.**  *Now we call applyCliqueReductions which is based on Proposition 16 as below. See Figure 3 for visualization.*

*1.*  *In (I) in Figure 3, we find that the derived curve of topLevelWeights is ABCD and F is strictly below it, so Item 3 in Proposition 16 is applicable and the corresponding vertex $z_{4}^{2}$ is removed.*
*2.*  *Because of the removal of $z_{4}^{2}$, the degrees of $z_{3}^{5}$, $z_{7}^{1}$ and $z_{8}^{5}$ are all decreased by 1, so their corresponding points on the coordinate plane are all left shift by 1 (see (II) in Figure 3). Notice that E, H, and B overlap at this time.*
*3.*  *Notice that G is strictly below the derived curved of topLevelWeights now, so we remove it like before, and this causes the left movement of H (see (III) in Figure 3).*
*4.*  *Analogously we remove $z_{8}^{5}$ because H is strictly below ABCD now (see (IV) in Figure 3).*
*5.*  *Note that removing $z_{3}^{5}$ is not allowed by Proposition 16, but it is permitted by Proposition 5. This shows the weakness of our* *applyCliqueReductions* *procedure, and we will address this issue in Section 3.3.*

Obviously, in order to perform effective reductions, we want $\omega_{1},\cdots ,\omega_{t}$ to be as big as possible. Hence, in Algorithm 2, we will try to increase their values. Furthermore, Proposition 16 is helpful in proving Proposition 17 below which computes a lower-bound of the cost of a feasible coloring.

**Proposition 17.**  *Given any feasible coloring S for G and $\mathrm{topLevelWeights}=\langle \omega_{1},\cdots ,\omega_{t}\rangle$, we have $\mathrm{cost}\left(S,G\right)\geq \Sigma_{i=1}^{t}\omega_{i}$.*

**Proof.**  See Appendix F.    □

Also, we have a proposition below which will be helpful in Section 3.3.

**Proposition 18.**  *Right before the execution of Line 22 in Algorithm 1, there do not exist any two cliques $C_{1},C_{2}$ s.t. $C_{1},C_{2}\in \mathrm{criticalCliqSet}$ and $C_{1}⊑C_{2}$.*

**Proof.**  See Appendix G.    □

### 3.2. Searching for Better Cliques

Given $\mathrm{topLevelWeights}=\langle \omega_{1},\cdots ,\omega_{t}\rangle$, Algorithm 2 attempts to increase the values of $\omega_{1},\cdots ,\omega_{t}$ and it even tries to find a clique whose size is bigger than *t*. So if Algorithm 2 completes, it will be able to confirm the following.

1. Each component in topLevelWeights has achieved its maximum possible value.
2. There exists no clique whose size is greater than |topLevelWeights|.

In Algorithm 2, we use updated(i) to denote whether $\omega_{i}$ is increased in the iteration for *i*. In Line 2, updated(i−1)=false means that we fail to update $\omega_{i-1}$. In our algorithm, there are two tricks that refer to updated(i) as below.

1. If $\omega_{i}=\omega_{i-1}$ and we have confirmed that there are no cliques that improve $\omega_{i-1}$, then there will be no cliques which improve $\omega_{i}$.
2. If $\omega_{i}=\omega_{i-1}$ and we fail to update $\omega_{i-1}$, then it will be hard for us to update $\omega_{i}$ as well, so we adopt a continue statement here to avoid probably hopeless efforts.

We also call the procedure applyCliqueReductions which was explained in the previous subsection. Notice that in Line 4, we enumerate maximal cliques which contain vertices in candSet only. To be specific, when i≤t, we will do so by considering vertices with weights greater than $\omega_{i}$ only, because we are now focusing on increasing $\omega_{i}$. Like the counterpart in Algorithm 1, we will shuffle related vertices randomly before each enumeration.

#### 3.2.1. Increasing Top Level Weights

Like Algorithm 1, we also exploit depth-first search to enumerate maximal cliques. Yet different from it, we will rarely enumerate *all* such maximal cliques. Instead, once we have found a clique that increases any value among $\omega_{1},\cdots ,\omega_{t}$, we will *immediately* perform reductions and break the enumeration procedure (see Line 14). Below we have a proposition that illustrates a sufficient and necessary condition in which $\omega_{i}\left(1\leq i\leq t\right)$ will be increased.

**Proposition 19.**  *As to the outermost loop in Algorithm 2, for any 1≤i≤t+1, $\omega_{i}$ will be increased if there exists a clique C⊆candSet s.t. |C|≥i.*

**Figure 4:**
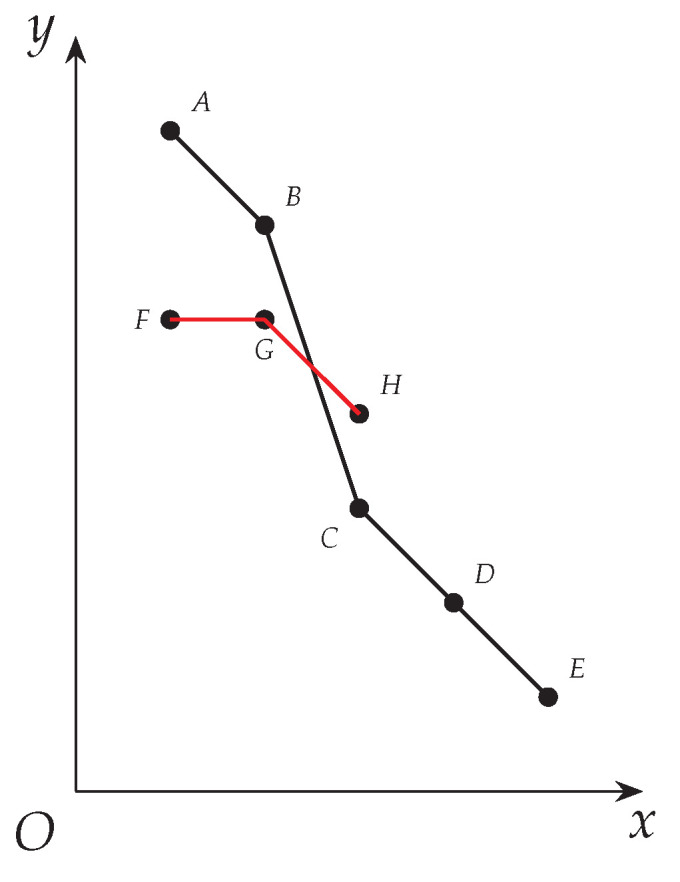
A clique is found to improve $\omega_{3}$. ABCDE represents topLevelWeights while FGH represents the derived curve of δ(C).

**Example 8.**  *Suppose we have topLevelWeights=〈7,6,3,2,1〉, and we are now focusing on increasing $\omega_{3}$ whose current value is 3. Suppose among vertices with weights greater than $\omega_{3}$, we have found a clique C with $\delta \left(C\right)=\langle w_{1},w_{2},w_{3}\rangle =\langle 5,5,4\rangle$ whose derived curve deviates above that of topLevelWeights at 3 (see FGH and ABCDE in Figure 4). So $\omega_{3}$ can now increase to be 4, and we will start another iteration to check whether $\omega_{3}$ can further increase.*

Notice that Line 14 breaks the clique enumeration loop and the program control of this algorithm will eventually be returned to Line 3 with an increased $\omega_{i}$. We do this for the following reason: Since we have increased $\omega_{i}$, any vertices that have a weight bigger than the previous $\omega_{i}$ but not bigger than the current $\omega_{i}$ will not help further increase $\omega_{i}$. Hence, we eliminate these vertices from candSet and enumerate maximal cliques again with respect to the same *i* (see Line 14). With a smaller candSet, we can increase $\omega_{i}$ to its maximum possible value more efficiently. In a word, we increase $\omega_{i}$ gradually until it reaches its maximum. Notice that Line 7 might also increase *t*, so long as the algorithm has found a clique that is bigger than any that have been found. Last we remind readers that although we are focusing on increasing $\omega_{i}$, there could be side effects that we increase $\omega_{i+1},\cdots ,\omega_{l}$ as well where l≤t, so long as we have found a clique that contains sufficiently many vertices with big weights.

#### 3.2.2. Effects of Better Cliques

There is a chance that the clique *C* obtained in Line 4 is not a maximal clique for the whole graph *G*, thus there may exist another clique in *G* that is a superset of *C* and has more reduction power. Alternatively, *C* may expand to a bigger clique by including vertices with a weight not greater than $\omega_{i}$ and lead to more reductions. Yet this is not a problem. If such a case exists, the full reduction power will be exploited in later iterations.

In the first few iterations of the outermost loop, *i* is relatively small and thus $\omega_{i}$ is relatively big, which is likely to result in a relatively small candSet, so enumerating cliques in candSet probably costs relatively little time. Moreover, these cliques may lead to effective reductions which significantly decrease the time cost of later enumerations. When i=t+1, we have candSet=V(G), thus in the worst case, *we will have to enumerate all maximal cliques in G*, which seems to be time-consuming and thus infeasible. Yet this is not so serious, because

1. we are dealing with large sparse graphs which often obey the power-distribution law,
2. and we have performed considerable reductions before, so at this time, *G* is likely to be small enough to allow maximal clique enumerations. In Section 3.4, we will also set a cutoff for enumerating cliques.

Last we remind readers that as *i* increases and $\omega_{i}$ decreases, candSet becomes larger and larger, and thus enumerating cliques will become more and more time-consuming, so we need to set a cutoff for enumerations (see Section 3.4). Due to this cutoff, once we fail to confirm that $\omega_{i}$ has achieved its maximum, we will not make any effort to confirm whether $\omega_{j}$ has arrived at its best possible value for any j>i.

Moreover, we have a proposition below which shows that, given sufficient run time, Algorithm 2 will be able to increase $\omega_{i}$ to its maximum possible value for any 1≤i≤ω(G), where ω(G) is the maximum size of a clique in *G*.

**Proposition 20.**  *As to the outermost loop in Algorithm 2, we have*

*1.*  *for any 1≤i≤t, right before i is increased by 1, there exist no cliques which deviate above topLevelWeights at i.*
*2.*  *for i=t+1, when the iteration ends, there exist no cliques which deviate above topLevelWeights at i.*

Then by this proposition, we have a theorem below which shows that our clique reduction algorithm is as effective as the counterpart which enumerates all maximal cliques in *G*, if time permits. To describe this theorem we first define the equality relation between two lists in Definition 9.

**Definition 9.**  *Given two list of weights $L_{1}=\langle \omega_{1}^{1}\cdots \omega_{t_{1}}^{1}\rangle$ and $L_{2}=\langle \omega_{1}^{2}\cdots \omega_{t_{2}}^{2}\rangle$ we say that $L_{1}=L_{2}$ iff $t_{1}=t_{2}$ and $\omega_{i}^{1}=\omega_{i}^{2}$ for any $1\leq i\leq t_{1}=t_{2}$.*

**Theorem 1.**  *Let $L_{1}$ be the topLevelWeights returned after Algorithms 1 and 2 are executed successively, and $L_{2}$ be the topLevelWeights returned after Algorithm 4 is executed, then $L_{1}=L_{2}$.*

Note that Algorithm 4 can be time-consuming even for sparse graphs.
**Algorithm 4:** computeTopLevelWeightsWithBF(*G*)
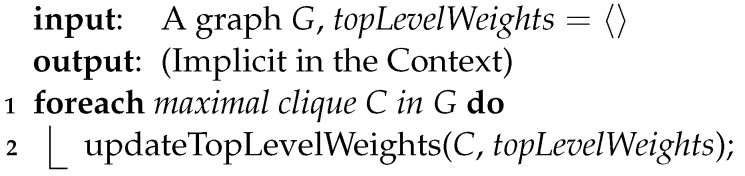


### 3.3. Post Reductions

Section 3.1 mentions that we have not fully exploited Proposition 5 to perform reductions, so in this subsection, we deal with the remaining case. At this stage, for each vertex, we will examine whether it is absorbed by some certain clique in criticalCliqSet and perform reductions if so.

### 3.4. Implementation Issues

Although we apply various tricks to enumerate diverse cliques for effective reductions, our algorithm may still become stuck in dense subgraphs, so we have to set a certain cutoff for our algorithm.

We believe that a good reduction algorithm should not focus too much on a local subgraph, so our cutoff will prevent each clique enumeration from spending too much time. The impact of this compromise is that we have to sacrifice some good properties above, to be specific, we now cannot expect that all $\omega_{i}$ values in topLevelWeights will increase to their maximum. Yet in our parameter setting, there are still quite a few $\omega_{i}$ values that are confirmed to achieve their optimum.

Furthermore, in some large graphs, we may need to consider a great many vertices and enumerate cliques that contain them, so there could be numerous enumerations. Hence, even though each enumeration needs a small amount of time, the total time cost of so many enumerations might not be affordable, so we also need to limit the total amount of time spent on enumerations.

#### 3.4.1. Limiting The Number of Decisions Made in Each Enumeration

Notice that we adopt a depth-first search to enumerate maximal cliques in Algorithms 1 and 2. During each depth-first enumeration, decisions of whether a vertex should be included in the current clique have to be made, and the search has to traverse both branches recursively, so there may be an exponential number of decisions for a single depth-first enumeration. Hence, in any enumeration, if topLevelWeights has been unable to be improved within λ consecutive decisions, we will simply stop this enumeration and go on to the next one.

#### 3.4.2. Limiting Running Time

Some benchmark graphs contain a large number of vertices that are of the greatest weights or degrees, so there can be a great amount of enumerations in Algorithm 1. Moreover as to Algorithm 2, there can be many candidate vertices that may form a clique to improve a particular component in topLevelWeights, hence numerous enumerations may be performed as well.

Even though we limit the number of decisions and thus limit the time spent in each enumeration, too many enumerations may still cost our algorithm so much time. Hence in practice, we employ another parameter *T* to limit the running time of our algorithm. More specifically in Algorithm 2, we will check whether the total time spent from the very beginning of our whole algorithm is greater than *T*. If so we will simply stop Algorithm 2 and turn to post reductions.

In fact, if Algorithm 2 is stopped because of this parameter, there can be cases as below. For the sake of presentation, we let *K* be the number of components in topLevelWeights which is equal to the size of the greatest clique that has been found.

1. Algorithm 2 is unable to tell whether there exists a clique *C* s.t. |C|≤K and *C* is able to improve a particular component in topLevelWeights.
2. Algorithm 2 has confirmed that any clique containing at most *K* vertices will not improve topLevelWeights. Yet it is unable to confirm whether there exists a clique whose size is bigger than *K*.

#### 3.4.3. Programming Tricks

In graph algorithms, there is a common procedure as follows. Given a graph and its two vertices *u* and *v*, determine whether *u* and *v* are neighbors. In our program, this procedure is called frequently, so we have to implement it efficiently. However, it is unsuitable to store large sparse graphs by adjacency matrices. Therefore, we adopted a hash-based data structure which was proposed in [18] to do so.

In our algorithm, we often have to obtain vertices of certain weights or degrees. Moreover, as vertices are removed, the degrees of their neighbors will be decreased. Furthermore, our algorithm interleaves between clique sampling and graph reductions, which requires us to maintain such relations in time. So we need efficient data structures to maintain vertices of each degree and/or weight in the reduced graph. Hence, we adapted the so-called Score-based Partition in [19] to do so.

## 4. Related Works

To our best knowledge, the only algorithm on reductions for vertex weighted coloring is RedLS [11], and its details are shown in Algorithm 5. In this algorithm, *C* is a candidate clique being constructed and each vertex in candSet is connected to each one in *C*. Hence, any single vertex in candSet can be added into *C* to form a greater clique.
**Algorithm 5:** RedLS
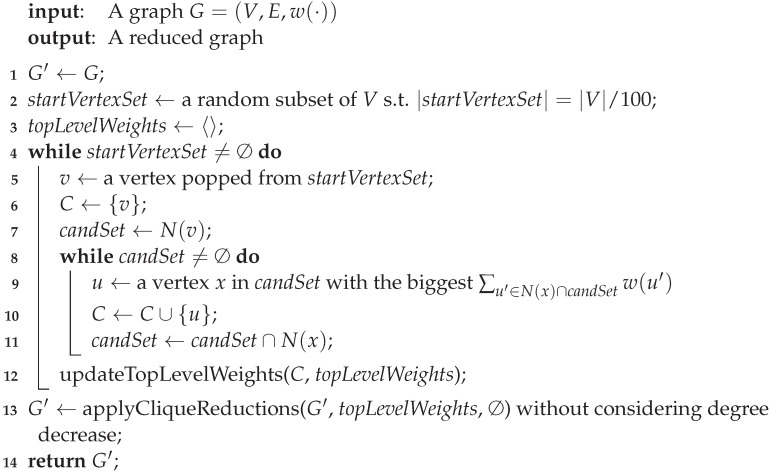


In Line 2, 1% of the vertices in *V* are randomly collected to obtain startVertexSet. As to the outer loop starting from Line 4, each vertex like *v* in startVertexSet is picked and a maximal clique containing *v* is constructed from Lines 6 to 11, based on a heuristic inspired by FastWClq [12]. In the inner loop starting from Line 8, Line 9 picks a vertex *u* in candSet, Line 10 places the vertex *u* into *C*, and Line 11 eliminates vertices which are not connected to every one in *C*, i.e., which are impossible to be added into *C* to make greater cliques.

Notice that Line 9 selects a next vertex to put into *C* with some look-head technique. To be specific, rather than choose the heaviest vertices and maximize current benefits, it tries to maximize the total weight of the remaining possible vertices, i.e., N(x)∩candSet, with a hope for greater future benefits. So given a vertex *v*, Algorithm 5 always aims to look for maximum or near-maximum weight cliques that contain it.

Each time a maximal clique *C* is constructed, Algorithm 5 will compare topLevelWeights with *C* and updates topLevelWeights if needed (see Line 12). Actually RedLS adopts the so-called ‘relaxed’ vertex partition, yet the effects are equivalent to our descriptions with topLevelWeights in Algorithm 5. After enumerating cliques with respect to vertices in startVertexSet, Algorithm 5 will call the applyCliqueReductions procedure and perform reductions based on Proposition 16. However, when determining whether a vertex namely *u* can be removed, it always takes d(u) in the whole graph as *u*’s degree, i.e., no degree decrease will be taken into account. In a nutshell, RedLS consists of clique sampling and graph reductions as successive procedures, which is different from our interleaving approach.

## 5. Experiments

We will present solvers and benchmarks, parameter settings, presentation protocols, results, and discussions in this section.

### 5.1. Solvers and Benchmarks

We consider a list of networks online that were accessed via http://networkrepository.com/ on 1 January 2018. They were originally unweighted, and to obtain the corresponding MinVWC instances, we use the same method as in [11,12]. For the *i*-th vertex $v_{i}$, $w\left(v_{i}\right)=\left(i\;\mathrm{mod}\;200\right)+1$. For the sake of space, we do not report results on graphs with fewer than 100,000 vertices or fewer than 1,000,000 edges. There is an instance named soc-sinaweibo which contains 58,655,849 vertices and 261,321,033 edges and thus is too large for our program, so our program ran out of memory and we do not report its result. In the following experiments, we simply disable the local search component in RedLS [11] and compare its reduction method to our algorithm.

Our algorithm was coded in Java and open source via https://github.com/Fan-Yi/iterated-clique-reductions-in-vertex-weighted-coloring-for-large-sparse-graphs accessed on 1 June 2023. It was compiled by OpenJDK 20.0.1 and run in an OpenJDK 64-bit Server VM (build 20.0.1+9-29, mixed mode, sharing). The experiments were conducted on a workstation with Intel(R) Xeon(R) Platinum 8260 CPU @ 2.40GHz CPU with 266 GB RAM under CentOS 7.9. Since we shuffle vertices in Algorithms 1 and 2, there exists randomness in the effectiveness of reduction. Yet we only test one arbitrary seed, since the benchmark graphs are diverse and each of them contains a large number of maximal cliques.

### 5.2. Parameter Settings

As to the parameter λ that limits the number of branching decisions in each depth-first enumeration procedure, we set it as $10,000*d_{\max}$ where $d_{\max}$ is the maximum degree in the input graph. On the other hand, RedLS was run with the default parameter setting in the machine environment reported by [11]. In fact, RedLS usually completes reductions in a significantly shorter time compared to our algorithm, yet this is not a big problem, because this paper focuses only on the potential effectiveness of a reduction algorithm, instead of its efficiency. Since the MinVWC problem is NP-hard, even a small number of additionally removed vertices may decrease a great amount of later search time, so our idea is meaningful.

As to the parameter *T* that limits the total running time of enumerations, we set it as 1200 s.

### 5.3. Presentation Protocols

For each instance, we report the number of vertices and edges in the original graph (denoted by ‘Original’ in Table 1) as well as that obtained by RedLS and our algorithm (denoted by ‘RedLS-reduced’ and ‘ours’, respectively, in the same table). In Table 1, we mainly compare the number of remaining vertices obtained by RedLS and that by our algorithm (Columns 4 and 6), and better results (smaller numbers) are shown in **bold**.

To show the effectiveness of our algorithm more clearly, we also report the percentage of remaining vertices, $\rho =|V^{\prime }\left|/|V\right|$, where *V* is the set of original vertices and $V^{\prime }$ is the set of remaining vertices after reductions. So the closer ρ is to 0, the more effective our algorithm is. Furthermore, the time column reports the number of seconds needed by our algorithm to perform reductions.

### 5.4. Main Results and Discussions

From Table 1, we observe the following.

1. Our algorithm obtains significantly better results in most of these instances compared to RedLS. Among all the graphs, the number of remaining vertices returned by RedLS is at least 10,000. However, on nearly 20% of the instances, our algorithm returns a result less than 10,000. Moreover, on more than 10% of the instances, it returns a result less than 1000.
2. On more than 40% of the instances, our percentage of remaining vertices is smaller than 10%, while on nearly 20%, the respective results are smaller than 1%.
3. The most attractive result lies in the road-net category, in which our algorithm returned subgraphs that contained 156, 86, 14, and 54 vertices, respectively, with |E| slightly more than |V|. However, RedLS returns subgraphs that contain at least 800,000 vertices. Thanks to our algorithm, it seems that optimal solutions for these graphs can now be easily found by state-of-the-art complete solvers.

### 5.5. Individual Impacts

We will show individual impacts of our three successive procedures as well as the optimality of top-level weights returned.

#### 5.5.1. Individual Impacts of Our Successive Procedures

To show that each of our three successive procedures is necessary, we calculate the number of vertices removed in each procedure during the execution of our algorithm. In Table 2, we use $\Delta_{1}$, $\Delta_{2}$ and $\Delta_{3}$ to represent the number of vertices removed by Algorithms 1 and 2 and post reductions, respectively. We select representative instances from most categories in order to reflect the individual impacts comprehensively.

From this table, we find that Algorithm 2 may sometimes remove no vertices, and post reductions usually have great contributions, which is why we allow equations to hold and extend statements in [11] to present Definition 3.

#### 5.5.2. Optimality of Top Level Weights

Finally, we discuss the optimality of topLevelWeights returned by our algorithm which will play an essential role in future works. Notice that Algorithm 2 tries to enumerate all possible cliques that may increase any component of topLevelWeights and even attempt to find a clique whose size is bigger than |topLevelWeights|. In practice, Algorithm 2 was able to confirm that some particular $\omega_{i}$ values had achieved their maximum. To be specific, we take instances web-it-2004, sc-pwtk and delaunay_n24 as examples, and show our experimental results in this aspect as below.

1. As to web-it-2004, our experiment guaranteed that each $\omega_{i}$ value had achieved its maximum and there existed no clique whose size was bigger than |topLevelWeights|. This is the best result which ensures that no better top-level weights can be found. This also implies that we have found the smallest number of remaining vertices. No better results can be obtained by clique reductions. In Table 1, all such instances are marked with ∗ in our |V| column.
2. As to sc-pwtk, our experiment guaranteed that each $\omega_{i}$ value had achieved its maximum, but it was unable to tell whether a clique with a size greater than |topLevelWeights| existed. In this sense, future works on this instance can focus on finding a clique of greater size.
3. As to delaunay_n24, our experiment could only make certain that the first two $\omega_{i}$ values of topLevelWeights returned had achieved their maximum, but there were still two components that were not confirmed. Hence, more efforts are to be made in this instance.

## 6. Conclusions

In this paper, we have proposed an iterated reduction algorithm for the MinVWC problem based on maximal clique enumerations. It alternates between clique sampling and graph reductions and consists of three successive procedures: promising clique reductions, better-bound reductions and post reductions. Experimental results on several large sparse graphs show that the effectiveness of our algorithm significantly outperforms that of RedLS in most of the instances. Moreover, it makes a big improvement on about 10% to 20% of them, especially on the road-net instances. Also, we have shown and discussed individual impacts as well as practical properties of our algorithm. Last we have a theorem that indicates that our algorithm’s reduction effects are equivalent to that of a counterpart which enumerates all maximal cliques in the input graph if time permits.

However, our clique enumeration procedures are somewhat brute-force, which may waste a great amount of time checking useless cliques. Furthermore given a vertex, clique reductions assume that each of its neighbors has a distinct color, yet this is not always the case and thus may limit the power of reductions.

For future works, we will develop various heuristics to sample promising cliques that are both effective and efficient for reductions. Also, we plan to develop reductions that allow neighbors of a vertex to have repeated colors.

## Figures and Tables

**Table 1:** Reductions on instances. Those numbers of remaining vertices that are confirmed to be optimal are marked with ‘*’.

Graph	Original		RedLS-Reduced		Ours		Performances	
	|V|	|E|	|V|	|E|	|V|	|E|	ρ	**Time**
dbpedia-link	11,621,692	78,621,046	**1,966,025**	27,556,529	2,668,018	66,546,584	0.2296	3668.492
delaunay_n22	4,194,304	12,582,869	4,148,216	5,174,821	**4,146,955**	12,440,822	0.9887	7566.296
delaunay_n23	8,388,608	25,165,784	8,296,041	1,359,491	**8,292,928**	24,878,744	0.9886	4469.89
delaunay_n24	16,777,216	50,331,601	16,593,030	8,182,952	**16,586,824**	49,760,425	0.9887	17,185.211
friendster	8,658,744	45,671,471	1,361,582	1,121,756	**1,315,470**	27,948,658	0.1519	2921.834
hugebubbles-00020	21,198,119	31,790,179	**21,198,117**	2,576,473	21,198,119	31,790,179	1.0000	1520.533
hugetrace-00010	12,057,441	18,082,179	**12,057,439**	698,458	12,057,441	18,082,179	1.0000	949.587
hugetrace-00020	16,002,413	23,998,813	**16,002,411**	4,245,244	16,002,413	23,998,813	1.0000	1165.196
inf-europe_osm	50,912,018	54,054,660	8,164,188	6,592,211	**156**	257	0.0000	2629.477
inf-germany_osm	11,548,845	12,369,181	2,272,030	19,285,716	86${}^{*}$	135	0.0000	10,502.181
inf-roadNet-CA	1,957,027	2,760,388	1,443,067	16,681,503	14${}^{*}$	18	0.0000	155.53
inf-roadNet-PA	1,087,562	1,541,514	812,126	12,885,609	54${}^{*}$	85	0.0000	64.437
inf-road-usa	23,947,347	28,854,312	8,850,794	2,227,418	**1588**	2523	0.0001	1205.464
rec-dating	168,792	17,351,416	138,886	11,540,694	**136,335**	17,294,606	0.8077	1213.253
rec-epinions	755,761	13,396,042	600,037	3,356,797	**551,260**	12,728,686	0.7294	3784.631
rec-libimseti-dir	220,970	17,233,144	188,610	1,235,483	**178,847**	17,114,097	0.8094	1310.625
rgg_n_2_23_s0	8,388,608	63,501,393	5,504,561	2,630,246	**60,568**	511,433	0.0072	8026.464
rgg_n_2_24_s0	16,777,216	132,557,200	12,163,095	2,654,218	**86,152**	771,045	0.0051	6891.269
rt-retweet-crawl	1,112,702	2,278,852	183,284	105,537,956	**108,136**	889,563	0.0972	2786.033
sc-ldoor	952,203	20,770,807	909,666	6,529,032	**909,407**	20,768,557	0.9551	1227.66
sc-msdoor	415,863	9,378,650	404,759	19,430,909	**404,697**	9,377,124	0.9731	1728.423
sc-pwtk	217,891	5,653,221	216,906	121,200,597	**216,256**	5,627,582	0.9925	4844.375
sc-rel9	5,921,786	23,667,162	5,921,770	11,333,101	**5,921,723**	23,667,036	1.0000	2205.027
sc-shipsec1	140,385	1,707,759	108,500	1,254,967	12,040${}^{*}$	238,889	0.0858	135.641
sc-shipsec5	179,104	2,200,076	99,518	1,555,956	16,316${}^{*}$	316,735	0.0911	255.063
soc-buzznet	101,163	2,763,066	61,740	4,821,649	**49,386**	2,485,394	0.4882	1235.923
soc-delicious	536,108	1,365,961	105,134	18,412,125	**35,441**	386,667	0.0661	2120.439
soc-digg	770,799	5,907,132	114,210	65,106,957	**74,049**	4,061,847	0.0961	1232.244
soc-dogster	426,820	8,543,549	**194,311**	249,959	213,189	7,262,428	0.4995	1439.012
socfb-A-anon	3,097,165	23,667,394	772,099	10,122,746	**636,050**	18,094,936	0.2054	1301.598
socfb-B-anon	2,937,612	20,959,854	636,188	1,738,199	**513,959**	15,708,329	0.1750	1380.445
socfb-uci-uni	58,790,782	92,208,195	3,858,784	678,005	**1,409,601**	6,743,138	0.0240	2423.396
soc-flickr	513,969	3,190,452	71,625	26,730,529	**43,056**	2,147,398	0.0838	1220.377
soc-flickr-und	1,715,255	15,555,041	174,859	11,028,549	**170,039**	12,382,047	0.0991	1302.161
soc-flixster	2,523,386	7,918,801	221,304	24,174,416	**112,261**	2,616,450	0.0445	1380.599
soc-FourSquare	639,014	3,214,986	109,565	40,074,246	**78,979**	1,769,972	0.1236	1640.654
soc-lastfm	1,191,805	4,519,330	219,171	19,999,513	**146,684**	2,258,601	0.1231	4018.428
soc-livejournal	4,033,137	27,933,062	262,009	2,552,908	**136,522**	3,924,419	0.0339	1459.076
soc-livejournal-user-groups	7,489,073	112,305,407	3,117,769	21,348	**2,995,174**	106,475,490	0.3999	10,996.874
soc-LiveMocha	104,103	2,193,083	83,955	6,651,778	**63,514**	2,018,567	0.6101	1288.405
soc-ljournal-2008	5,363,186	49,514,271	**232,350**	59,042,551	666,701	24,572,530	0.1243	1502.869
soc-orkut-dir	3,072,441	117,185,083	**2,114,644**	27,556,529	2,649,700	114,727,342	0.8624	2247.88
soc-orkut	2,997,166	106,349,209	**2,193,033**	5,174,821	2,568,364	103,885,482	0.8569	2005.389
soc-pokec	1,632,803	22,301,964	961,510	1,359,491	**755,949**	17,670,190	0.4630	25,267.249
soc-twitter-higgs	456,631	12,508,442	283,106	1,121,756	**256,087**	11,116,984	0.5608	1542.861
soc-youtube	495,957	1,936,748	127,815	2,576,473	**42,031**	793,160	0.0847	1446.011
soc-youtube-snap	1,134,890	2,987,624	184,961	698,458	**57,846**	1,015,713	0.0510	3398.715
tech-as-skitter	1,694,616	11,094,209	314,778	4,245,244	**203,445**	4,374,731	0.1201	1915.585
tech-ip	2,250,498	21,643,497	646,599	6,592,211	**445,389**	18,033,323	0.1979	11,419.378
twitter_mpi	9,862,152	99,940,317	**801,988**	19,285,716	1,346,594	80,524,123	0.1365	5100.926
web-arabic-2005	163,598	1,747,269	18,352	16,681,503	436${}^{*}$	15,910	0.0027	354.495
web-baidu-baike	2,141,300	17,014,946	426,588	12,885,609	**396,647**	10,610,619	0.1852	2057.537
web-it-2004	509,338	7,178,413	28,302	2,227,418	1064${}^{*}$	197,532	0.0021	676.35
web-uk-2005	129,632	11,744,049	39,696	11,540,694	500${}^{*}$	124,750	0.0039	332.751
web-wikipedia2009	1,864,433	4,507,315	153,776	3,356,797	**8020**	145,183	0.0043	1493.974
web-wikipedia-growth	1,870,709	36,532,531	**833,848**	1,235,483	1,583,733	35,829,670	0.8466	874.927
web-wikipedia_link	2,936,413	86,754,664	**151,707**	2,630,246	756,853	50,293,310	0.2577	24,039.262
wikipedia_link_en	27,154,756	31,024,475	888,520	2,654,218	**510,753**	20,830,732	0.0188	8034.773

**Table 2:** Individual Impacts of Three Successive Procedures.

Instance	$\Delta_{1}$	$\Delta_{2}$	$\Delta_{3}$	Instance	$\Delta_{1}$	$\Delta_{2}$	$\Delta_{3}$
bn-human-BNU_1_0025865_session_1-bg	1,212,744	85	7433	sc-shipsec1	126,939	628	778
ca-hollywood-2009	672,443	103	5131	socfb-A-anon	2,440,611	1380	19,124
delaunay_n22	47,090	0	259	socfb-uci-uni	57,015,654	0	365,527
friendster	7,296,915	5737	40,622	soc-livejournal-user-groups	4,467,520	1972	24,407
inf-roadNet-CA	1,941,528	5653	9832	tech-as-skitter	1,475,837	0	15,334
rt-retweet-crawl	992,569	6378	5619	web-wikipedia2009	1,837,696	8391	10,326

## Data Availability

Not applicable.

## Appendix A. Proof of Proposition 1

**Proof.** 

1. (By contradiction) Assume that ${S|}_{G}$ is not a feasible coloring for G[U], then there exists an edge {x,y} in G[U] s.t. $c_{x}=c_{y}$. Since x,y∈U⊂V and G[U] is an induced subgraph of *G*, we have {x,y} is in *G*. This in turn implies that there exists an edge {x,y} in *G* s.t. $c_{x}=c_{y}$, which contradicts the precondition that ${S|}_{G}$ is a feasible coloring for *G*.
2. Suppose that ${S|}_{G}=\left\{\langle 1,V_{1}\rangle ,\cdots ,\langle k,V_{k}\rangle \right\}$, then considering that U⊂V, we have $\mathrm{cost}\left(S{|}_{G},G\left[U\right]\right)=\sum_{i=1}^{k}\max_{v\in V_{i}\cap U}w\left(v\right)\leq \sum_{i=1}^{k}\max_{v\in V_{i}\cap V}w\left(v\right)=\mathrm{cost}\left(S{|}_{G},G\right)$.

□

## Appendix B. Proof of Proposition 2

**Proof.** 

1. Case 1: j=k+1, so $V_{j}=\left\{x\right\}$.
  $$\begin{array}{ccc} \; & cost(S,G[V\setminus \{x\}])\\ \; & =\sum_{i=1}^{k}\max_{v\in V_{i}\cap \left(V\setminus \left\{x\right\}\right)}w\left(v\right)\\ \; & <\sum_{i=1}^{k}\max_{v\in V_{i}\cap \left(V\setminus \left\{x\right\}\right)}w\left(v\right)+w\left(x\right)\\ \; & =\sum_{i=1}^{k}\max_{v\in V_{i}\cap \left(V\setminus \left\{x\right\}\right)}w\left(v\right)+\max_{v\in V_{j}\cap V}w\left(v\right)\\ \; & =\sum_{i=1}^{k}\max_{v\in V_{i}\cap V}w\left(v\right)+\max_{v\in V_{j}\cap V}w\left(v\right) & \left(\sin\mathrm{ce}\;x\notin \overset{k}{\underset{i=1}{⋃}}V_{i}\right)\\ \; & =cost(S⊎\left(c_{x}\leftarrow j\right),G). \end{array}$$
2. Case 2: 1≤j≤k.
  $$\begin{array}{cc} \; & cost(S,G[V\setminus \{x\}])\\ \; & =\sum_{i=1}^{k}\max_{v\in V_{i}\cap \left(V\setminus \left\{x\right\}\right)}w\left(v\right)\\ \; & =\sum_{i=1\wedge i\neq j}^{k}\max_{v\in V_{i}\cap \left(V\setminus \left\{x\right\}\right)}w\left(v\right)+\max_{v\in V_{j}\cap \left(V\setminus \left\{x\right\}\right)}w\left(v\right)\\ \; & \leq \sum_{i=1\wedge i\neq j}^{k}\max_{v\in V_{i}\cap \left(V\setminus \left\{x\right\}\right)}w\left(v\right)+\max_{v\in (V_{j}\cup \left\{x\right\})\cap V}w\left(v\right)\\ \; & =\sum_{i=1\wedge i\neq j}^{k}\max_{v\in V_{i}\cap V}w\left(v\right)+\max_{v\in (V_{j}\cup \left\{x\right\})\cap V}w\left(v\right)\\ \; & \leq cost(S⊎\left(c_{x}\leftarrow j\right),G). \end{array}$$

□

## Appendix C. Proof of Proposition 3

**Proof.** Given any solution ${S|}_{G[W]}$ for G[W], we have there exists an extension to ${S|}_{G[W]}$, denoted by ${S|}_{G[U]}$, such that ${S|}_{G[U]}$ is feasible for G[U] and

$$cost\left(S{|}_{G[W]},G\left[W\right]\right)=cost\left(S{|}_{G[U]},G\left[U\right]\right).$$

Also for the same reason, given any solution ${S|}_{G[U]}$ for G[U], we have there exists an extension to ${S|}_{G[U]}$, denoted by ${S|}_{G}$, such that ${S|}_{G}$ is feasible for *G* and

$$cost\left(S{|}_{G[W]},G\left[W\right]\right)=cost\left(S{|}_{G},G\right).$$

Combining the statements above we have, given any solution ${S|}_{G[W]}$ for G[W], there exists an extension to ${S|}_{G[W]}$, denoted by ${S|}_{G}$, such that ${S|}_{G}$ is feasible for *G* and

$$cost\left(S{|}_{G[W]},G\left[W\right]\right)=cost\left(S{|}_{G},G\right).$$

□

## Appendix D. Proof of Proposition 4

(1) Since G[U] is a VWC-reduced subgraph of *G*, we have there exists an extension to $S^{*}{|}_{G[U]}$, denoted by ${\tilde{S}}^{*}{|}_{G}$, such that ${\tilde{S}}^{*}{|}_{G}$ is feasible for *G* and

$$\mathrm{cost}\left(S^{*}{|}_{G[U]},G\left[U\right]\right)=\mathrm{cost}\left({\tilde{S}}^{*}{|}_{G},G\right).$$

Now we prove by contradiction that ${\tilde{S}}^{*}{|}_{G}$ must be an optimal solution for *G*. Assume that ${\tilde{S}}^{*}{|}_{G}$ is not an optimal solution for *G*, then there exists a feasible coloring ${\overset{ˇ}{S}}^{*}{|}_{G}$ for *G* s.t. $\mathrm{cost}\left({\tilde{S}}^{*}{|}_{G},G\right)>\mathrm{cost}\left({\overset{ˇ}{S}}^{*}{|}_{G},G\right)$ and thus

$$\mathrm{cost}\left(S^{*}{|}_{G[U]},G\left[U\right]\right)=\mathrm{cost}\left({\tilde{S}}^{*}{|}_{G},G\right)>\mathrm{cost}\left({\overset{ˇ}{S}}^{*}{|}_{G},G\right),$$

i.e.,

$$\mathrm{cost}\left(S^{*}{|}_{G[U]},G\left[U\right]\right)>\mathrm{cost}\left({\overset{ˇ}{S}}^{*}{|}_{G},G\right).$$

Since ${\overset{ˇ}{S}}^{*}{|}_{G}$ is a feasible coloring for *G*, by Proposition 1, we have ${\overset{ˇ}{S}}^{*}{|}_{G}$ is also a feasible coloring for G[U] and

$$\mathrm{cost}\left({\overset{ˇ}{S}}^{*}{|}_{G},G\left[U\right]\right)\leq \mathrm{cost}\left({\overset{ˇ}{S}}^{*}{|}_{G},G\right).$$

This in turn implies that there exists a feasible coloring ${\overset{ˇ}{S}}^{*}{|}_{G}$ for G[U] and

$$\mathrm{cost}\left({\overset{ˇ}{S}}^{*}{|}_{G},G\left[U\right]\right)\leq \mathrm{cost}\left({\overset{ˇ}{S}}^{*}{|}_{G},G\right)<\mathrm{cost}\left(S^{*}{|}_{G[U]},G\left[U\right]\right),$$

which contradicts that $S^{*}{|}_{G[U]}$ is an optimal solution for G[U]. Alternatively we have ${\tilde{S}}^{*}{|}_{G}$ is an optimal solution for *G*.

(2) Based on the statements above, we have the following. Given any optimal solution $S^{*}{|}_{G[U]}$ for G[U], there exists an extension to $S^{*}{|}_{G[U]}$, denoted by ${\tilde{S}}^{*}{|}_{G}$, which is an optimal solution for *G* and

$$\mathrm{cost}\left(S^{*}{|}_{G[U]},G\left[U\right]\right)=\mathrm{cost}\left({\tilde{S}}^{*}{|}_{G},G\right).$$

Suppose $S^{↓}{|}_{G}$ is an arbitrary extension to $S^{↓}{|}_{G[U]}$ which is a coloring for *G*. Then by Proposition 2, we have

$$\mathrm{cost}\left(S^{↓}{|}_{G[U]},G\left[U\right]\right)\leq \mathrm{cost}\left(S^{↓}{|}_{G},G\right).$$

Because $S^{↓}{|}_{G}$ is an arbitrary extension, we cannot write ‘=’. Also because $S^{↓}{|}_{G[U]}$ is a non-optimal solution for G[U], we have

$$\mathrm{cost}\left(S^{*}{|}_{G[U]},G\left[U\right]\right)<\mathrm{cost}\left(S^{↓}{|}_{G[U]},G\left[U\right]\right).$$

Hence,

$$\mathrm{cost}\left({\tilde{S}}^{*}{|}_{G},G\right)=\mathrm{cost}\left(S^{*}{|}_{G[U]},G\left[U\right]\right)<\mathrm{cost}\left(S^{↓}{|}_{G[U]},G\left[U\right]\right)\leq \mathrm{cost}\left(S^{↓}{|}_{G},G\right).$$

This in turn implies that $S^{↓}{|}_{G}$ is not an optimal solution for *G*.

## Appendix E. Proof of Proposition 5

**Proof.** Let $C=\{v_{1},\cdots ,v_{\left|C\right|}\}$ s.t. $w\left(v_{1}\right)\geq \cdots \geq w\left(v_{\left|C\right|}\right)$. Suppose ${S|}_{G[V\setminus \{u\}]}$ is any feasible coloring for G[V∖{u}]. Now we are to prove that there exists an extension to ${S|}_{G[V\setminus \{u\}]}$, denoted by ${S|}_{G}$, such that ${S|}_{G}$ is feasible for the whole graph *G* and

$$\mathrm{cost}\left(S{|}_{G[V\setminus \{u\}]},G\left[V\setminus \left\{u\right\}\right]\right)=\mathrm{cost}\left(S{|}_{G},G\right).$$

Since *u* is absorbed by *C*, we have u∉C and d(u)<|C|, thus we have d(u)+1≤|C| and $v_{1},$⋯,$v_{d(u)+1}\in C\subseteq V\setminus \left\{u\right\}$. Now we construct

$${S|}_{G}{=S|}_{G[V\setminus \{u\}]}⊎\left(c_{u}\leftarrow c_{z}\right),$$

where

$$z\in \left\{v_{1},\cdots ,v_{d(u)+1}\right\}\subseteq C\subseteq V\setminus \left\{u\right\}.$$

So ${S|}_{G}$ is an extension to ${S|}_{G[V\setminus \{u\}]}$ by coloring *u* an existing color in ${S|}_{G[V\setminus \{u\}]}$.

1. Since *u* is distinct from $v_{1},\cdots ,v_{d(u)+1}$, we have assigning *u* a certain color among those of $v_{1},\cdots ,v_{d(u)+1}$ is possible. (We cannot assign *u* a color of itself, which is meaningless.)
2. Since *C* is a clique, we have $c_{v_{i}}\neq c_{v_{j}}$ for any 1≤i≠j≤|C|. Moreover because C⊆V∖{u}, we have there must be at least |C|>d(u) colors in any feasible coloring for G[V∖{u}]. For coloring *u*’s neighbors, the number of colors in use is at most d(u), hence, at least one color among those of $v_{1},\cdots ,v_{d(u)+1}$ is not in use. So we can use it to color *u* with causing any conflicts, and thus make ${S|}_{G}$ is a feasible coloring for *G*.
3. Considering that
  $$w\left(u\right)\leq w\left(v_{d(u)+1}\right)\leq \cdots \leq w\left(v_{1}\right),$$
  we have
  $$\mathrm{cost}\left(S_{G},G\right)=\mathrm{cost}\left(S{|}_{G[V\setminus \{v\}]},G\left[V\setminus \left\{v\right\}\right]\right).$$

□

## Appendix F. Proof of Proposition 17

**Proof.** (By contradiction) Assume that $\mathrm{cost}\left(S,G\right)<\Sigma_{i=1}^{t}\omega_{t}$. Now we are to show that *S* is not a feasible coloring which will contradict the preconditions. Without loss of generality, suppose $S=\{\langle 1,V_{1}\rangle ,\cdots ,\langle k,V_{k}\rangle \}$ is a coloring for *G* s.t. $\max_{v\in V_{1}}w\left(v\right)$≥⋯≥$\max_{v\in V_{k}}w\left(v\right)$, then we have k≥t by Proposition 16 and also

$$\mathrm{cost}\left(S,G\right)=\sum_{i=1}^{k}\max_{v\in V_{i}}w\left(v\right)<\Sigma_{i=1}^{t}\omega_{t}.$$

Since w(v)>0 for any v∈V and $\omega_{i}>0$ for any 1≤i≤t, we have there exists at least one 1≤l≤t s.t. $\max_{v\in V_{l}}w\left(v\right)<\omega_{l}$ and thus $\max_{v\in V_{k}}w\left(v\right)\leq \cdots \leq \max_{v\in V_{l}}w\left(v\right)<\omega_{l}$. By Proposition 16, we have there exists a clique $Q=\{v_{1},\cdots ,v_{\left|Q\right|}\}$ s.t. |Q|=l and

$$w\left(v_{1}\right)\geq \cdots \geq w\left(v_{\left|Q\right|}\right)=\omega_{l}>\max_{v\in V_{l}}w\left(v\right)\geq \cdots \geq \max_{v\in V_{k}}w\left(v\right).$$

Therefore,

$$v_{1},\cdots ,v_{\left|Q\right|}\notin ⋃V_{i=l}^{k}$$

and thus

$$v_{1},\cdots ,v_{\left|Q\right|}\in ⋃V_{i=1}^{l-1}.$$

Considering that

|Q|=l,

by the Pigeonhole Principle, we have there exists at least one 1≤s≤l−1 s.t. $V_{s}$ contains two or more vertices among $v_{1},\cdots ,v_{\left|Q\right|}$, that is, *S* is not a feasible coloring for *G*, which contradicts the preconditions. Alternatively we have proved that $\mathrm{cost}\left(S,G\right)\geq \Sigma_{i=1}^{t}\omega_{i}$. □

## Appendix G. Proof of Proposition 18

**Proof.** The proof includes two cases.

1. *$C_{1}$ enters criticalCliqSet first.* If $C_{1}⊑C_{2}$, then $C_{1}$ will be removed from criticalCliqSet at Line 14 before $C_{2}$ enters criticalCliqSet, i.e., they will not be in criticalCliqSet simultaneously.
2. *$C_{2}$ enters criticalCliqSet first.* If $C_{1},C_{2}\in \mathrm{criticalCliqSet}$, i.e., $C_{1}$ enters criticalCliqSet later, then by Proposition 15, we have $C_{1}⋢C_{2}$.

□
